# Redox regulation by reversible protein *S*-thiolation in bacteria

**DOI:** 10.3389/fmicb.2015.00187

**Published:** 2015-03-16

**Authors:** Vu Van Loi, Martina Rossius, Haike Antelmann

**Affiliations:** Institute of Microbiology, Ernst-Moritz-Arndt-University of GreifswaldGreifswald, Germany

**Keywords:** oxidative stress, protein *S*-thiolation, thiol-redox buffer, glutathione, bacillithiol, mycothiol

## Abstract

Low molecular weight (LMW) thiols function as thiol-redox buffers to maintain the reduced state of the cytoplasm. The best studied LMW thiol is the tripeptide glutathione (GSH) present in all eukaryotes and Gram-negative bacteria. Firmicutes bacteria, including *Bacillus* and *Staphylococcus* species utilize the redox buffer bacillithiol (BSH) while Actinomycetes produce the related redox buffer mycothiol (MSH). In eukaryotes, proteins are post-translationally modified to *S*-glutathionylated proteins under conditions of oxidative stress. *S*-glutathionylation has emerged as major redox-regulatory mechanism in eukaryotes and protects active site cysteine residues against overoxidation to sulfonic acids. First studies identified *S*-glutathionylated proteins also in Gram-negative bacteria. Advances in mass spectrometry have further facilitated the identification of protein *S*-bacillithiolations and *S*-mycothiolation as BSH- and MSH-mixed protein disulfides formed under oxidative stress in Firmicutes and Actinomycetes, respectively. In *Bacillus subtilis*, protein *S*-bacillithiolation controls the activities of the redox-sensing OhrR repressor and the methionine synthase MetE *in vivo*. In *Corynebacterium glutamicum*, protein *S*-mycothiolation was more widespread and affected the functions of the maltodextrin phosphorylase MalP and thiol peroxidase (Tpx). In addition, novel bacilliredoxins (Brx) and mycoredoxins (Mrx1) were shown to function similar to glutaredoxins in the reduction of BSH- and MSH-mixed protein disulfides. Here we review the current knowledge about the functions of the bacterial thiol-redox buffers glutathione, bacillithiol, and mycothiol and the role of protein *S*-thiolation in redox regulation and thiol protection in model and pathogenic bacteria.

## Introduction

The cytoplasm is a reducing environment and protein thiols are maintained in their reduced state by low molecular weight (LMW) thiol-redox buffers and enzymatic thiol-disulfide oxidoreductases, including the thioredoxin and glutaredoxin systems (Fahey, 2013; Van Laer et al., 2013). In their natural environment or during infections, bacteria encounter different reactive species, such as reactive oxygen, nitrogen, chlorine, and electrophilic species (ROS, RNS, RCS, RES) (Antelmann and Helmann, 2011; Gray et al., 2013a). These reactive species cause different post-translational thiol-modifications in proteins and activate or inactivate specific transcription factors resulting in expression of detoxification pathways. LMW thiol-redox buffers function in detoxification of different reactive species and are often present in millimolar concentrations in the cytoplasm.

The best studied LMW thiol is glutathione (GSH) present in eukaryotes and Gram-negative bacteria (Fahey, 2013). Most Gram-positive bacteria do not produce GSH. Instead, the Actinomycetes utilize mycothiol (MSH) as thiol-redox buffer (Jothivasan and Hamilton, 2008; Newton et al., 2008). In *Bacillus megaterium*, *Bacillus cereus*, and *Staphylococcus aureus*, coenzyme A (CoASH) serves as an abundant LMW thiol (Newton et al., 1996). Many Firmicutes bacteria, including *Bacillus* and *Staphylococcus* species utilize bacillithiol (BSH) and cysteine as major thiol-redox buffers (Newton et al., 2009). Alternative LMW thiols include also the betaine-histidine derivative ergothioneine that compensates for the absence of MSH in *Mycobacterium smegmatis mshA* mutants (Ta et al., 2011). Cysteine is used for alternative *S*-thiolations in the absence of BSH and MSH in *Bacillus subtilis* and *Corynebacterium glutamicum* since *S*-cysteinylated proteins were identified in *bsh* and *msh* mutants (Chi et al., 2011, 2014).

The protozoa Leishmania and Trypanosoma produce the glutathione-derivative trypanothione (bis-glutathionyl-spermidine or TSH_2_). In *Escherichia coli*, glutathionylspermidine (GSP) was detected during the stationary phase (Fahey, 2013). Some microaerophilic γ-proteobacteria utilize glutathione amide (GASH) which forms a persulfide (GASSH) during photoautotrophic growth on high concentrations of sulfide (Bartsch et al., 1996).

Under conditions of oxidative stress, LMW thiols form mixed disulfides with protein thiols which is termed protein *S*-thiolation. In eukaryotes, protein *S*-glutathionylation has emerged as major redox-regulatory mechanism that controls the activity of redox sensing transcription factors and protects active site Cys residues against irreversible oxidation to sulfonic acids (Dalle-Donne et al., 2009). *S*-glutathionylation controls numerous physiological processes, such as cellular growth and differentiation, cell cycle progression, transcriptional activity, cytoskeletal function, cellular metabolism, and apoptosis (Klatt and Lamas, 2000; Ghezzi, 2005, 2013; Dalle-Donne et al., 2007, 2009). *S*-glutathionylation must meet several criteria to function as redox-control mechanism: (1) reversibility, (2) specificity to active site Cys, (3) change in protein function/activity, and (4) induction by ROS or RNS. *S*-glutathionylation serves as a form of GSH storage to prevent the export of GSSG under oxidative stress (Dalle-Donne et al., 2009). Many eukaryotic proteins, like α-ketoglutarate dehydrogenase, glyceraldehyde 3-phosphate dehydrogenase, ornithine δ-aminotransferase, pyruvate kinase, heat specific chaperones, and regulatory proteins (c-Jun, NF-κB) are reversibly inactivated or activated by *S*-glutathionylation (Dalle-Donne et al., 2009; Kehr et al., 2011). However, the regulatory role of protein *S*-thiolation for bacterial physiology has only recently been investigated. Here we review the current knowledge about the functions of the bacterial redox buffers GSH, MSH, and BSH and their roles for protein *S*-thiolations in GSH-, MSH- and BSH-producing bacteria.

## Sources of reactive oxygen, electrophile, and chlorine species (ROS, RES, RCS)

**Figure 1:**
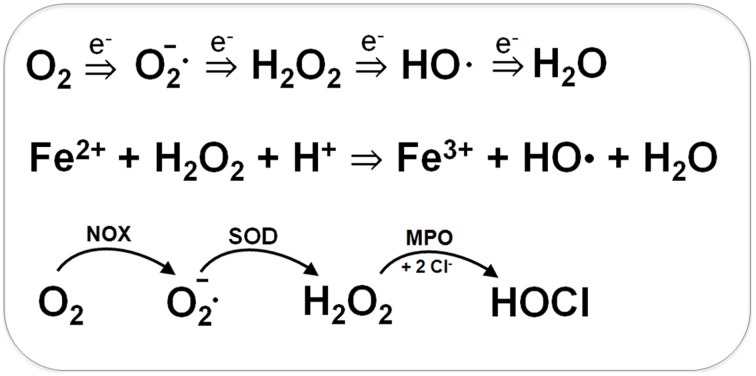
**Generation of Reactive Oxygen Species (ROS) during respiration and HOCl production by activated neutrophils during infections**. ROS are generated in bacteria during respiration by stepwise one-electron transfer to O_2_ producing superoxide anion, hydrogen peroxide and hydroxyl radical. The highly reactive hydroxyl radical is also produced from H_2_O_2_ and Fe^2+^ in the Fenton reaction. During infections, activated neutrophils generate superoxide anion by the NADPH oxidase (NOX) that is converted to H_2_O_2_ by the superoxide dismutase (SOD). Myeloperoxidase (MPO) is released upon degranulocytosis producing the highly reactive hypochlorous acids (HOCl) from H_2_O_2_ and Cl^−^ as potent killing agent for pathogenic bacteria.

Bacteria encounter ROS during respiration or by the oxidative burst of activated neutrophils during infections (Imlay, 2003, 2008, 2013). The incomplete stepwise reduction of molecular oxygen (O_2_) leads to generation of superoxide anions (O_2_•^−^), hydrogen peroxide (H_2_O_2_) and the highly reactive hydroxyl radical (OH•) (Figure 1). Superoxide anion and H_2_O_2_ are also produced by autoxidation of flavoenzymes (Mishra and Imlay, 2012; Imlay, 2013). Superoxide dismutases (SOD) convert O_2_•^−^ to H_2_O_2_. Several peroxide scavenging enzymes, such as catalases and peroxidases catalyze the detoxification of H_2_O_2_. H_2_O_2_ reacts with ferrous iron (Fe^2+^) in the Fenton reaction generating the highly toxic hydroxyl radical (OH•) which can damage all cellular macromolecules (Imlay, 2003, 2008). H_2_O_2_ destroys the Fe-S-cluster of dehydratases and inactivates single ferrous iron-centers of redox enzymes (Mishra and Imlay, 2012; Imlay, 2013).

During the oxidative burst, activated neutrophils release O_2_•^−^, H_2_O_2_, nitric oxide (NO), and hypochlorous acid (HOCl) with the aim to kill invading pathogenic bacteria (Forman and Torres, 2001; Winterbourn and Kettle, 2013). The neutrophil NADPH oxidase (NOX) shuttles electrons from NADPH to O_2_ in the phagosomal lumen and generates around 20 μM superoxide anion. Myeloperoxidase (MPO) is released upon degranulation in millimolar concentrations. MPO catalyzes the dismutation of O_2_•^−^ to H_2_O_2_ and subsequent conversion of H_2_O_2_ and chloride to HOCl (Figure 1) (Winterbourn and Kettle, 2013). NO is generated in neutrophils by the inducible nitric oxide synthase (iNOS) catalyzing the oxidation of L-arginine to L-citrulline. The reaction of NO with O_2_•^−^ leads to formation of peroxynitrite (ONOO^−^). Thus, neutrophils release ROS, RNS, and the highly reactive HOCl as antimicrobial defense mechanism.

**Figure 2:**
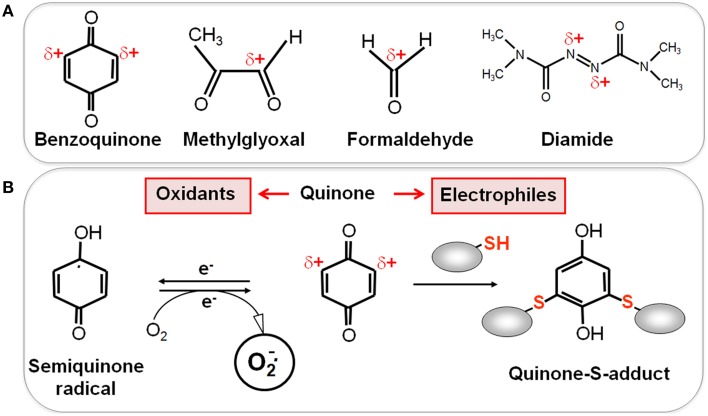
**Reactive Electrophilic Species (RES) with partial positive charges (δ+) (A) and the reaction of quinones with thiols via the *S*-alkylation and oxidation chemistry (B). (A)** In quinones and aldehydes the electrons are drawn to carbonyl oxygen leaving the partial positive charges at neighboring carbon atoms that become electrophilic. Diamide is an electrophilic azocompound that causes disulfide stress. **(B)** Quinones can react as electrophiles with the nucleophilic thiol group of Cys residues via thiol-*S*-alkylation leading to irreversible thiol-*S*-adduct formation. In the oxidative mode, quinones are incompletely reduced to semiquinone radicals that generate superoxide anions and can oxidize protein thiols to disulfides.

Reactive electrophilic species (RES) have electron-deficient centers and can react with the nucleophilic Cys thiol group via the thiol-*S*-alkylation chemistry (Figure 2) (Antelmann and Helmann, 2011). RES include quinones, aldehydes, epoxides, diamide and α,β-unsaturated dicarbonyl compounds. RES are often generated as secondary reactive intermediates from oxidation products of amino acids, lipids or carbohydrates (Marnett et al., 2003; Rudolph and Freeman, 2009). Quinones are lipid-electron carriers of the respiratory chain, including ubiquinone and menaquinone (Farrand and Taber, 1974). Soil bacteria encounter quinones as redox active components of humic substances and in dissolved organic matter (Ratasuk and Nanny, 2007). The toxic dicarbonyl compound methylglyoxal is produced in all organisms from triose-phosphate intermediates as byproduct of the glycolysis and can be generated also from amino acids metabolism (Ferguson et al., 1998; Booth et al., 2003; Kalapos, 2008). Bacteria also have to cope with the carbonyl compound formaldehyde. Formaldehyde is an intermediate in the C_1_-metabolism of methanotrophic and methylotrophic bacteria and is ubiquitously distributed in the environment. Thus, bacteria have evolved conserved pathways for detoxification of formaldehyde and methylglyoxal that involve LMW thiols.

In eukaryotic cells, RES are implicated in many pathophysiological processes and modulate signaling pathways (Mackay and Knock, 2015). Eukaryotic cells produce lipid-derived RES, such as malondialdehyde (MDA) and 4-hydroxy-2-nonenal (HNE) (Rudolph and Freeman, 2009). HNE is generated from polyunsaturated fatty acids in biological membranes by a radical-based peroxidation chain reaction (Jacobs and Marnett, 2010). Furthermore, 15-deoxy-Δ12,14-prostaglandin J2 (15d-PGJ2) is generated from arachidonic acid during inflammation and 2-trans-hexadecenal (2-HD) is produced during sphingolipid metabolism which promotes apoptosis (Wang et al., 2014). Bacterial membrane lipids also contain unsaturated fatty acids which are synthesized at higher levels during adaptation to cold shock to maintain the fluidity of the membrane (De Mendoza, 2014). These unsaturated fatty acids in bacterial membrane lipids could be the target for ROS leading to lipid peroxidation products in bacteria. Lipid hydroperoxides, such as linoleic acid hydroperoxide are sensed by the redox-sensing MarR-type repressor OhrR. OhrR regulates the peroxiredoxin OhrA that functions in detoxification of organic hydroperoxides (Atichartpongkul et al., 2001; Fuangthong et al., 2001). However, the fatty acid-derived peroxidation product which is sensed by OhrR *in vivo* remains to be identified.

## Post-translational thiol-modifications of proteins by ROS, RES, and RCS in bacteria

**Figure 3:**
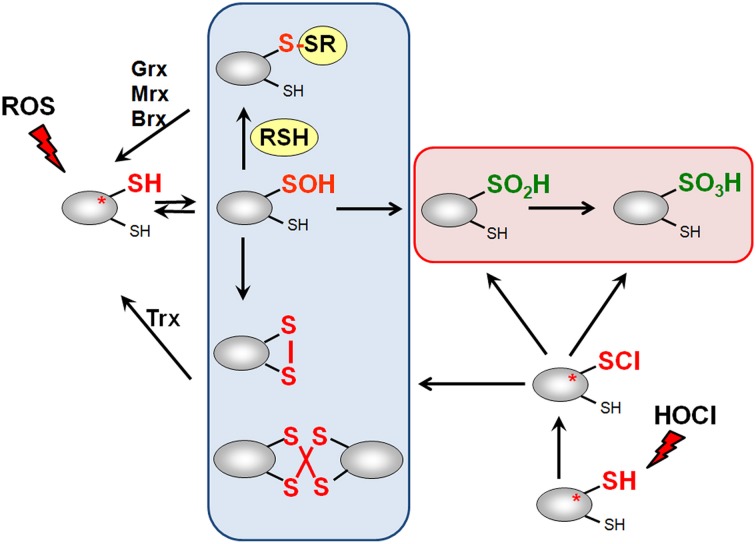
**Thiol-chemistry of ROS and HOCl with thiol-containing proteins**. The Cys thiol group is oxidized by ROS to an unstable Cys sulfenic acid intermediate (Cys-SOH) that reacts further with proximal thiols to form intramolecular and intermolecular disulfides or mixed disulfides with LMW thiols (RSH), such as glutathione, bacillithiol or cysteine, termed as *S*-thiolations. HOCl leads to chlorination of protein thiols to sulfenylchloride intermediates (Cys-SCl) that react further to form disulfides. In the absence of proximal thiols, the chlorinated Cys is overoxidized to Cys sulfinic and sulfonic acids. Disulfides function as redox switches to control protein activity and protect thiol groups against overoxidation to Cys sulfinic and sulfonic acids.

ROS, RES, and RCS can damage all cellular macromolecules including proteins, nucleic acids or carbohydrates (Imlay, 2008, 2013; Jacobs and Marnett, 2010; Gray et al., 2013a). However, in eukaryotes low levels of ROS and RES act also as second messengers to modulate signal transduction pathways (Rudolph and Freeman, 2009; Mackay and Knock, 2015). Bacterial transcription factors often use redox-sensitive Cys residues for sensing of ROS, RES, and RCS to control the expression of specific detoxification pathways (Antelmann and Helmann, 2011; Gray et al., 2013a). The thiol group of cysteine is subject to reversible and irreversible post-translational thiol-modifications that lead to inactivation or activation of the transcription factor. Protein thiols can be reversibly oxidized to protein disulfides and irreversibly overoxidized to sulfinic or sulfonic acids by ROS (Antelmann and Helmann, 2011). ROS lead first to oxidation of protein thiols to Cys sulfenic acids as unstable intermediates (R-SOH) (Figure 3). Cys sulfenic acid rapidly reacts further with other thiols to form intramolecular and intermolecular protein disulfides or mixed disulfides with LMW thiols, collectively termed as *S*-thiolations (e.g., *S*-cysteinylations, *S*-glutathionylations, *S*-mycothiolations, and *S*-bacillithiolations). Protein *S*-thiolations protect the thiol groups against the irreversible overoxidation to Cys sulfinic (R-SO_2_H) and sulfonic acid (R-SO_3_H). This is particularly important for essential and abundant proteins whose overoxidation would lead to loss of cell viability and requires new protein synthesis to replace inactivated proteins. However, eukaryotic sulfiredoxins are able to reduce sulfinic acids in 2-Cys peroxiredoxins, but sulfiredoxins are not present in bacteria (Lowther and Haynes, 2011).

Hypochloric acid (HOCl) is a strong two-electron oxidant and chlorinating agent with a high redox potential [*E*^0′^(HOCl/Cl^−^) = 1.28 mV] (Davies, 2011). HOCl targets most strongly the sulfur-containing amino acids cysteine and methionine with the second-order rate constant of *k* = 3 × 10^7^ M^−1^ s^−1^ (Hawkins et al., 2003). HOCl first chlorinates the thiol group to form the unstable sulfenylchloride intermediate that reacts further with another thiol group to disulfides. In the absence of another thiol, the chlorinated thiol group is overoxidized very rapidly to sulfinic or sulfonic acids (Hawkins et al., 2003) (Figure 3). We confirmed that strong disulfide stress responses are caused by HOCl in different Gram-positive bacteria *in vivo* and detected mixed protein disulfides with Cys, BSH, and MSH as major oxidation products (Chi et al., 2011, 2013, 2014).

RNS cause reversible thiol-modifications: nitric oxide (NO) leads to *S*-nitrosothiol formation (RS-NO) and peroxinitrite (ONOO^−^) causes *S*-nitrothiol (RS-NO_2_) formation. Alternatively, *S*-nitrosothiol (e.g., GSNO or MSNO) can be formed by direct reaction of NO with LMW thiols (Antelmann and Helmann, 2011).

RES can react via the thiol-S-alkylation chemistry with Cys thiols. However, quinones have two modes of action, an oxidation and an alkylation mode. In the oxidation mode, the one-electron reduction of quinones generates the highly reactive semiquinone radical leading to generation of superoxide anions (Figure 2). The electrophilic reaction of quinones involves the 1,4-reductive Michael-type addition of thiols to quinones (Marnett et al., 2003). Quinones lead to irreversible thiol-*S*-alkylation and protein aggregation to deplete protein thiols in the proteome *in vivo* (Liebeke et al., 2008). However, non-toxic quinone concentrations resulted in reversible thiol-disulfide switches in RES-sensing redox regulators (e.g., YodB, CatR, QsrR, NemR) to activate the expression of specific quinone detoxification pathways (Antelmann and Helmann, 2011; Gray et al., 2013a; Lee et al., 2013). Methylglyoxal reacts with nucleophilic centers of the DNA and with the amino acids arginine, lysine and cysteine causing advanced glycation end-products (Bourajjaj et al., 2003). The lipid-derived electrophiles MDA and HNE were shown to alkylate DNA bases and protein thiols leading to DNA and membrane damages in eukaryotes (Rudolph and Freeman, 2009).

## Biosynthesis and functions of major LMW thiol-redox buffers in bacteria

### Biosynthesis, uptake, and functions of glutathione in bacteria

**Figure 4:**
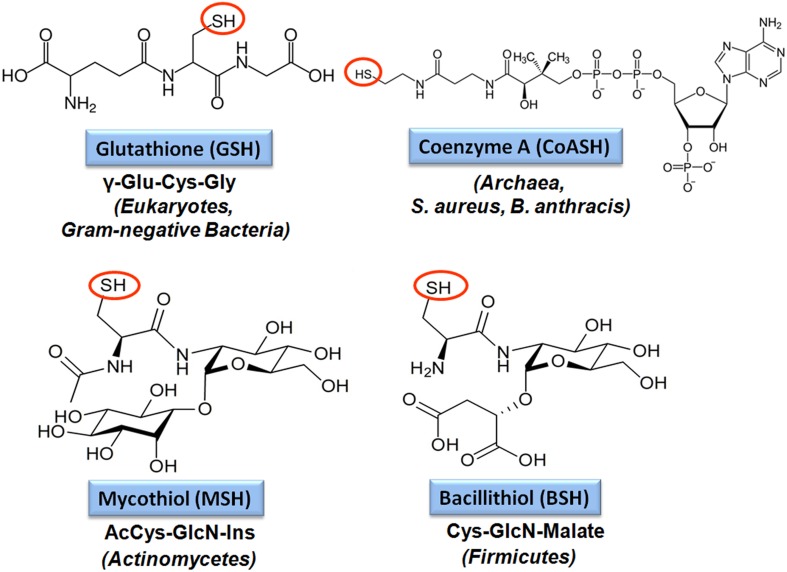
**Structures of major bacterial low molecular weight (LMW) thiols**. Major LMW thiols are glutathione (GSH) in eukaryotes and Gram-negative bacteria, mycothiol (MSH) in Actinomycetes and bacillithiol (BSH) in Firmicutes. Coenzyme A (CoASH) also serves as a LMW thiol-redox buffer in some bacteria, like in *S. aureus* and *B. anthracis*.

The tripeptide glutathione (γ-glutamylcysteinyl-glycine; GSH) is utilized as major LMW thiol-redox buffer in Gram-negative bacteria and in some Gram-positive Firmicutes bacteria, including *Streptococcus agalactiae*, *Listeria monocytogenes*, and *Clostridium acetobutylicum* (Figure 4). In *E. coli*, GSH biosynthesis occurs in two steps: The γ-glutamate cysteine ligase (GshA) catalyzes the formation of γ-glutamylcysteine (γ-Glu-Cys) from glutamate and cysteine. In the second step, ligation of glycine to γ-Glu-Cys is catalyzed by glutathione synthase (GshB) (Meister, 1995; Anderson, 1998). In *S. agalactiae* and *L. monocytogenes*, a bifunctional fusion protein GshF is present that exhibits both GshA and GshB activities (Gopal et al., 2005; Janowiak and Griffith, 2005). Interestingly, *Lactococcus lactis, Streptococcus pneumoniae* and *Haemophilus influenzae* do not synthesize GSH, but encode GSH-uptake mechanisms. In *S. pneumoniae*, the GSH-uptake from the host is mediated by the ABC transporter binding protein GshT (Potter et al., 2012; Vergauwen et al., 2013). In addition, the cystine importer TcyBC was shown to be primed for GSH import by GshT. In *H. influenzae*, GSH import is mediated by the ABC-transporter DppBCDF and requires the periplasmic GSH-binding protein GbpA (Vergauwen et al., 2010). Strikingly, these pathogens utilize host-derived GSH as protection mechanism against the host immune defense.

GSH is present in millimolar concentrations in the cytoplasm of *E. coli* (Masip et al., 2006; Fahey, 2013). GSH maintains protein thiols in the reduced state and serves as a storage form of cysteine. In contrast to cysteine, GSH is resistant to metal-catalyzed autooxidation because of its bound Cys amino and carboxyl groups that prevent ligation of heavy metal ions (Fahey, 2013). During the bacterial growth and under oxidative stress, GSH is oxidized to glutathione disulfide (GSSG). The NADPH-dependent glutathione reductase (Gor) keeps GSH in its reduced state to maintain a high GSH/GSSG ratio ranging from 30:1 to 100:1 in the cytoplasm. The standard thiol-disulfide redox potential of the GSH redox couple was calculated as *E*^0′^(GSSG/GSH) = −240 mV at physiological pH values (Hwang et al., 1995; Van Laer et al., 2013).

The various detoxification functions of GSH have been extensively studied in *E. coli gsh* mutants. In *E. coli*, GSH functions in detoxification of ROS, RES, RCS, RNS, xenobiotics, antibiotics, toxic metals, and metalloids (Masip et al., 2006) (Table 1). Detoxification of xenobiotics, electrophiles and antibiotics by GSH occurs either spontaneously by *S*-conjugation or by the catalytic activity of GSH-*S*-transferases (Fahey, 2013). The GSH-*S*-conjugates are usually excreted from the cell as non-toxic mercapturic acid derivatives. GSH was shown to function as a cofactor in methylglyoxal detoxification in *E. coli*. The major pathway for methylglyoxal detoxification in *E. coli* is the GSH-dependent glyoxalase pathway. The glyoxalase-I (GloA) catalyzes formation of *S*-lactoyl-GSH from GSH-hemithioacetal and glyoxalase-II (GloB) converts *S*-lactoyl-GSH to D-lactate (Ferguson et al., 1998; Booth et al., 2003). In addition, glyoxalase-III operates GSH-independently to convert methylglyoxal to lactate. The glyoxalase-I encoding *gloA* gene and the *nemRA* operon are redox-regulated by the NemR repressor under methylglyoxal, quinone and HOCl stress (Gray et al., 2013b; Lee et al., 2013; Ozyamak et al., 2013). The glyoxalase GloA and the oxidoreductase NemA are important for methylglyoxal survival and confer resistance to methylglyoxal in *E. coli* (Ozyamak et al., 2013). The resistance to methylglyoxal is also linked to the activation of potassium efflux by the *S*-lactoyl-GSH intermediate leading to cytoplasmic acidification (Ferguson et al., 1998; Booth et al., 2003). The cytoplasmic acidification limits the interaction of methylglyoxal with DNA bases. Thus, potassium efflux and detoxification by GloA protect against methylglyoxal toxicity in *E. coli*.

**Table 1:** **Functions of the bacterial redox buffers glutathione, bacillithiol, and mycothiol**.

**Redox buffer**	**Organism**	**Functions of thiol-redox buffers and thiol-dependent enzymes**	**References**
Glutathione	*Escherichia coli*	GSH functions in detoxification of ROS, RES, RCS, RNS, xenobiotics, antibiotics, toxic metals, metalloids	Masip et al., 2006 Potter et al., 2012
	*Salmonella* Typhimurium	Gor: GSSG reductase	
		Gpx: GSH-dependent peroxidase	
		Gst: GSH *S*-transferases required for conjugation of alkylating agents and antibiotics	
		Grx: Glutaredoxins for reduction of *S*-glutathionylated proteins	
		GloA/GloB: glyoxalase-I/II for GSH-dependent conversion of methylglyoxal to lactate	
Bacillithiol	*Bacillus subtilis*	BSH involved in detoxification of hypochlorite, diamide, methylglyoxal, ROS (paraquat, H_2_O_2_), alkylating agents and fosfomycin	Gaballa et al., 2010 Chi et al., 2011
	*Staphylococcus aureus*	BSH provides a Zn buffer for labile Zn pool	Ma et al., 2014
		YpdA: possible BSSB reductase	Gaballa et al., 2010
		FosB: BSH-dependent epoxide hydrolase for fosfomycin detoxification	Lamers et al., 2012 Roberts et al., 2013 Thompson et al., 2013, 2014
		YfiT/BstA: DinB-family BSH *S*-transferases required for conjugation of alkylating agents (monochlorobimane, 1-chloro-2,4-dinitrobenzene and cerulenin)	Newton et al., 2011 Perera et al., 2014
		BrxA/BrxB: Bacilliredoxins for reduction of *S*-bacillithiolated proteins	Gaballa et al., 2014
		GlxA/GlxB: glyoxalase-I/II for BSH-dependent conversion of methylglyoxal to lactate	Chandrangsu et al., 2014
Mycothiol	*Streptomyces coelicolor*	MSH protects against ROS, RES, NO, toxins, antibiotics (erythromycin, vancomycin, rifampicin), heavy metals, maleylpyruvate, ethanol, gentisate, glyphosate, arsenate in Actinomycetes	Newton et al., 2008 Fahey, 2013 Liu et al., 2013
	*Mycobacterium tuberculosis*	Mtr: MSSM reductase	
	*Corynebacterium glutamicum*	Tpx, AhpE, Mpx: MSH-dependent peroxidases	Chi et al., 2014 Hugo et al., 2014
		Mst: DinB-family MSH *S*-transferases required for conjugation of alkylating agents and antibiotics (monochlorobimane, DTNB, rifampicin, cerulenin)	Newton et al., 2012
		LmbT, LmbV and LmbE: MSH *S*-transferases for biosynthesis of the lincosamide antibiotic lincomycin in *S. lincolnensis*	Zhao et al., 2015
		Mca: *S*-conjugate amidase cleaves MSH-*S*-conjugates to mercapturic acids	Newton et al., 2008, 2011
		Mrx1: Mycoredoxin-1 for reduction of *S*-mycothiolations	Van Laer et al., 2012
		MscR/AdhE/FadH: MSNO reductase/ formaldehyde dehydrogenase	Newton et al., 2008 Lessmeier et al., 2013 Witthoff et al., 2013
		Cg3349: maleylpyruvate isomerase for maleylpyruvate detoxification in *C. glutamicum*	Feng et al., 2006
		ArsC1/C2: MSH-dependent arsenate reductases	Ordonez et al., 2009

Interestingly, expression of the *E. coli gshAB* genes in the industrial important *Clostridium acetobutylicum* enhances robustness and alcohol production as a promising strategy for engineering industrial production strains. Thus, GSH protects also against large-scale ethanol and butanol production in *C. acetobutylicum* during fermentation (Hou et al., 2013).

### Functions of glutathione in the virulence of pathogenic bacteria

GSH has many detoxification functions to maintain the redox balance of the cytoplasm, but only recently the role of GSH for the control of virulence functions has been explored in the pathogenic bacteria *S. pneumoniae*, *L. monocytogenes*, and *Salmonella* Typhimurium (Potter et al., 2012; Song et al., 2013; Reniere et al., 2015) (Table 2). In *S. pneumoniae*, the glutathione reductase Gor and the GSH-importer GshT were required for oxidative stress protection and metal ion resistance. Moreover, the *gshT* mutant was attenuated in colonization and invasion in a mouse model of pneumococcal infection (Potter et al., 2012). Thus, GSH protects against the host immune defense and contributes to fitness of *S. pneumoniae*.

**Table 2:** **The role of thiol-redox buffers for virulence in pathogenic bacteria**.

**Redox buffer**	**Organism**	**Genes for biosynthesis or uptake**	**Virulence phenotypes of mutants**	**References**
Glutathione (uptake)	*Streptococcus pneumoniae*	*gshT* (GSH importer)	*gshT* mutant attenuated in colonization and invasion in a mouse model of pneumococcal infection	Potter et al., 2012
Glutathione (biosynthesis and uptake)	*Listeria monocytogenes*	*gshF* (γ-Glu-Cys ligase/GSH synthase)	Virulence defect of the *gshF* mutant caused the lack of PrfA activation and lower *actA* expression by bacterial and host GSH	Reniere et al., 2015
Glutathione (biosynthesis)	*Salmonella* Typhimurium	*gshA* (γ-Glu-Cys ligase) *gshB* (GSH synthase)	*gshA* and *gshB* mutants attenuated in acute model of salmonellosis in NRAMP1^R^ mice; GSH protects against ROS and RNS produced by NOX in mice	Song et al., 2013
Bacillithiol (biosynthesis)	*Staphylococcus aureus*	*bshA* (glycosyltransferase) *bshB* (deacetylase) *bshC* (Cys ligase)	COL and USA300 *bshA* mutants impaired in human whole-blood survival assays; SH1000 natural *bshC* mutant survival defect in macrophage phagocytosis assays	Posada et al., 2014 Pöther et al., 2013
Mycothiol (biosynthesis)	*Mycobacterium tuberculosis*	*mshA1* (glycosyltransferase) *mshA2*(phosphatase) *mshB* (deacetylase) *mshC* (Cys ligase) *mshD* (acetyltransferase)	*mshC* mutant impaired in growth and survival in mouse model of infection	Sareen et al., 2003; Sassetti and Rubin, 2003

The intracellular pathogen *L. monocytogenes* is able to synthesize GSH via the *gshF* fusion protein, but GSH can be also imported from the host (Reniere et al., 2015). The *L. monocytogenes gshF* mutant was two-fold less virulent compared to the wild type in a mice model. In addition, the *gshF* mutant was sensitive to oxidative stress, contains lower levels of ActA and formed small plaques in tissue culture assays that measure cell-to-cell spread (Reniere et al., 2015). The Actin assembly-inducing protein ActA is controlled by the virulence regulator PrfA and used by *L. monocytogenes* to move through the host cells (Freitag et al., 2009). It was shown that the virulence phenotype of the *gshF* mutant is caused by the lack of PrfA activation by bacterial and host-derived GSH (Reniere et al., 2015). Interestingly, activation of PrfA is mediated by an allosteric binding of GSH to PrfA, but not by *S*-glutathionylation. Thus, GSH plays a role as signaling molecule to activate virulence gene expression in an intracellular pathogen.

In *S*. Typhimurium, GSH antagonizes the bacteriostatic effects of RNS *in vivo* and *gshA* mutants were sensitive to ROS and RNS (Song et al., 2013). Thus, GSH presents a first line defense against ROS and RNS produced by the host immune system. This was shown in an acute model of salmonellosis in mice expressing the wild-type NRAMP1^R^ allele (natural resistance-associated macrophage protein 1) which is linked to high NO production of the macrophages. The *gshA* and *gshB* mutants were attenuated in this acute model of salmonellosis. It was further shown that GSH protects against ROS and RNS produced by the NADPH phagocyte oxidase and inducible nitric oxide synthase (iNOS) in this mice model (Song et al., 2013). These recent studies highlight the important roles of GSH in the control of virulence functions, expression of virulence factors and pathogen fitness under infection conditions in *S. pneumoniae*, *L. monocytogenes*, and *S*. Typhimurium. As shown for *L. monocytogenes*, GSH might play similar roles to activate virulence gene expression by redox control of virulence gene regulators in other pathogens which remains to be elucidated.

### Redox proteomic methods to study protein *S*-glutathionylation at a global scale

**Figure 5:**
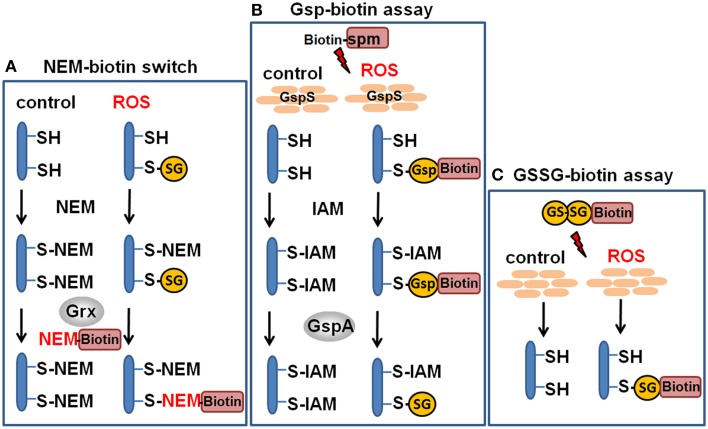
**Redox proteomics methods to study protein *S*-glutathionylation at a global scale**. Mass spectrometry-based methods for identification of *S*-glutathionylations include the glutaredoxin-coupled NEM-biotin switch assay **(A)**, the biotin-Gsp assay, **(B)** or the N,N-biotinyl glutathione disulfide (BioGSSG) assay, **(C)** (Lind et al., 2002; Brennan et al., 2006; Kehr et al., 2011; Zaffagnini et al., 2012a). In the biotin-Gsp assay, E. coli GspS is expressed in mammalian cells and converts GSH and biotinyl-spermine (biotine-spm) to biotin-glutathionylspermidine (biotin-Gsp). Proteins in ROS-treated cells are modified by biotin-Gsp-*S*-thiolation (Chiang et al., 2012; Lin et al., 2015). The biotin-spm is removed from the enriched biotin-Gsp-*S*-thiolated peptides by GspA and the GSS-peptides are identified by mass spectrometry.

Advances in probe design and mass spectrometry-based thiol-trapping approaches have facilitated the detection of specific reversible thiol-modifications, including sulfenylation, nitrosylation, glutathionylation, and sulfhydrations of proteins at a global scale (Leonard and Carroll, 2011; Pan and Carroll, 2013; Paulsen and Carroll, 2013; Gupta and Carroll, 2014; Zhang et al., 2014). Different methods have been applied for specific detection of *S*-glutathionylations in eukaryotic cells, including the use of GSH-specific antibodies and the labeling of *S*-thiolations with ^35^S-cysteine followed by 2D gel electrophoresis and phosphoimaging (Fratelli et al., 2002, 2003, 2004). However, the specificity of the GSH antibodies is questionable and the gel-based detection of *S*-thiolations using ^35^S-cysteine does not distinguish between *S*-cysteinylations and *S*-glutathionylations or other forms of *S*-thiolations. Hence, more sensitive mass spectrometry-based redox proteomics methods have been developed, including the glutaredoxin-coupled NEM-biotin switch assay and the treatment of cell extracts with N,N-biotinyl glutathione disulfide (BioGSSG) (Lind et al., 2002; Brennan et al., 2006; Kehr et al., 2011; Zaffagnini et al., 2012a) (Figure 5). Both methods make use of the specific streptavidin enrichment of biotinylated peptides that improve the identification of *S*-glutathionylated peptides using mass spectrometry. The NEM biotin-switch assay was successfully applied to detect protein *S*-glutathionylation in eukaryotic endothelial cells and malaria parasites which applies glutaredoxin for reduction of protein-SSG followed by NEM-biotin alkylation and enrichment using streptavidin columns (Lind et al., 2002; Kehr et al., 2011). The biotin-GSSG approach has been applied to identify *S*-glutathionylated proteins in the green algae *Chlamydomonas reinhardtii* (Zaffagnini et al., 2012a) and in the photosynthetic cyanobacterium *Synechocystis* sp. PCC6803 (Chardonnet et al., 2015). In total, 383 *S*-glutathionylated proteins were identified in *Synechocystis* sp. PCC6803 and 125 glutathionylation sites were mapped by mass spectrometry. In addition, the peroxiredoxin PrxII (Sll1621) and the 3-phosphoglycerate dehydrogenase PGDH (Sll1908) could be *S*-glutathionylated by BioGSSG *in vitro* (Chardonnet et al., 2015).

In another approach, biotinyl-spermine (biotine-spm) and *in vivo* expressed *E. coli* GspS have been applied for mammalian cells to convert GSH to biotin-glutathionylspermidine (biotin-Gsp) which subsequently modified proteins by Biotin-Gsp-*S*-thiolation (Chiang et al., 2012; Lin et al., 2015). The biotine-spm is removed enzymatically by GspA from the enriched biotin-Gsp-*S*-thiolated peptides and the GSS-peptides are identified by mass spectrometry. This approach allows the identification of *S*-glutathionylation sites without the biotin-tag and enhances the coverage for *S*-glutathionylated proteins. In mammalian cells, 1409 *S*-glutathionylated cysteines in 913 proteins were identified using the Gsp-biotin approach (Chiang et al., 2012; Lin et al., 2015). This makes the application of this chemoenzymatic approach using the GspS enzyme attractive for global and specific studies of *S*-glutathionylated proteins.

In *S*. Typhimurium, protein *S*-glutathionylation has been studied at a global scale by top-down and bottom-up proteomic approaches (Ansong et al., 2013). Top-down proteomics uses whole proteins for separation and fragmentation directly in the mass spectrometer. In bottom-up proteomics approaches proteins are digested by a protease and the peptide mixtures are analyzed by mass spectrometry to identify proteins at the peptide level. The top-down proteomic approach identified 563 proteins with 1665 post-translational modifications in *S*. Typhimurium. The authors identify 25 *S*-thiolated proteins in cells grown in complete LB medium including 16 *S*-glutathionylated proteins and nine *S*-cysteinylated proteins. Interestingly, a subset of nine *S*-glutathionylated are modified by *S*-cysteinylation in infection-like minimal LPM medium (Table 3). This could indicate a shift from *S*-glutathionylation to *S*-cysteinylation under infection-like conditions in *S*. Typhimurium. These *S*-thiolated proteins include phosphoglycerate kinase (Pgk), elongation factor (Tuf) and enolase (Eno) that are also targets for *S*-glutathionylations in endothelial cells (Fratelli et al., 2002). The top-down proteomics results were verified by bottom-up proteomics approaches to identify the specific *S*-thiolated Cys peptides (Ansong et al., 2013). Structural analysis revealed that *S*-glutathionylation occurred mostly at buried Cys residues and not at surface-exposed Cys. *S*-glutathionylation on buried Cys was also shown for the enolase whose activity is known to be modified by *S*-thiolation in human cells (Fratelli et al., 2002). It is postulated that *S*. Typhimurium switches from *S*-glutathionylation to *S*-cysteinylation during infection conditions as novel redox-control mechanism. In agreement with the proteome data, transcriptome results point to an up-regulation of Cys biosynthesis and down-regulation of GSH biosynthesis under infection-like conditions. However, the physiological role of this *S*-thiolation switch for redox control of the identified protein targets under ROS stress remains to be elucidated. Furthermore, no blocking of reduced thiols with NEM or IAM was performed to avoid artificial disulfide formation. Thus, it remains to be verified that the observed *S*-thiolations are not caused by artificial thiol-disulfide exchange. Previous studies have also shown that *L. monocytogenes* is able to both import and synthesize GSH (Reniere et al., 2015). Furthermore, the non-GSH-utilizing *S. aureus* was shown to import GSH during growth in LB medium (Pöther et al., 2013). Thus, the possible uptake of GSH in *S*. Typhimurium from LB-medium could contribute to the observed *S*-glutathionylations which needs to be investigated.

**Table 3:** **Targets for protein S-thiolation by bacterial thiol-redox buffers**.

**Redox buffer**	**Organism**	**Functions of *S*-thiolated proteins**	***S*-thiolated Cys**	**References**
Glutathione	*Salmonella* Typhimurium	16 protein-SSG and nine protein-SSCys identified in LB medium cultures nine protein-SSG in LB switch to protein-SSCys in minimal medium:		Ansong et al., 2013
		DnaK (chaperone)	Cys15	
		CspD (cold shock protein)	Cys19	
		HNS (transcription regulator)	Cys21	
		MinE (cell devision factor)	Cys16	
		Ndk (nucleoside diphosphate kinase)	Cys139	
		GrxC (glutaredoxin)	Cys66	
		RplC (50S ribosomal protein)	Cys199	
		YifE (unknown function)	Cys64	
		YjgF (translation inhibitor)	Cys107	
Glutathione	*Escherichia coli*	OxyR (peroxide sensor)	Cys199 redox-sensing	Kim et al., 2002
		Gap (glyceraldehyde-3-phosphate DH)	Cys152 active site	Brandes et al., 2009
		MetE (methionine synthase)	Cys645 not conserved	Hondorp and Matthews, 2004
		PpaC (PAPS reductase)	Cys239 active site	Lillig et al., 2003
Glutathione	*Neisseria meningitidis*	EstD (esterase)	Cys54 substrate binding	Chen et al., 2013
Glutathione	*Pseudo-alteromonas haloplanktis*	PhSOD (iron-superoxide dismutase)	Cys57 conserved	Castellano et al., 2008
Glutathione	*Synechocystis* sp. PCC6803	383 total protein-SSG 125*S*-glutathionylation sites:		Chardonnet et al., 2015
		Inorganic pyrophosphatase	Cys164	
		Phosphoribulokinase	Cys19	
		PAPS reductase	Cys230	
		Triose phosphate isomerase	Cys127	
		IMP dehydrogenase	Cys222	
		ADP-glucose pyrophosphorylase	Cys55	
		RubisCo	Cys422, Cys242	
		MerA (mercury reductase)	Cys78 active site	Marteyn et al., 2013
		AbrB (repressor of hydrogenase operon)	Cys34 redox-sensing	Cassier-Chauvat et al., 2014
Bacillithiol	*Bacillus subtilis*	54 total protein-SSB including eight conserved protein-SSB:		Chi et al., 2011 Chi et al., 2013
	*Bacillus pumilus*	MetE (methionine synthase)	Cys730 active site	
	*Bacillus amyloliquefaciens*	PpaC (Mn-dependent inorganic pyrophosphatase)	Cys158 active site	
		SerA (D-3-phosphoglycerate DH)	Cys410 conserved	
		AroA (chorismate mutase)	Cys126 conserved	
	*Staphylococcus carnosus*	TufA (Elongation factor Tu)	Cys83 GTP-binding site	
		GuaB (IMP dehydrogenase)	Cys308 active site	
		YphP/BrxA (bacilliredoxin)	Cys53 active site	
		YumC (Ferredoxin-NADP reductase2)	Cys85 active site	
Mycothiol	*Corynebacterium glutamicum*	25 total protein-SSM identified:		
		MalP (Maltodextrin phosphorylase)	Cys180 conserved	
		MetE (Methionine synthase)	Cys713 active site	
		Hom (Homoserine DH)	Cys239	
		Ino-1 (Myo-inositol-1-P-synthase)	Cys79	
		Fba (Fructose-bisphosphate aldolase)	Cys332	
		SerA (Phosphoglycerate DH)	Cys266	
		Pta (Phosphate acetyltransferase)	Cys367	
		XylB (pentulose/hexulose kinase	Cys338	
		GuaB1/2 (IMP dehydrogenase)	Cys302/Cys317 active site	
		NadC (Nicotinate-nucleotide pyrophosphorylase)	Cys114	
Mycothiol	*Corynebacterium glutamicum*	PurL (Phosphoribosyl formylglycinamidine synthase)	Cys716	Chi et al., 2014
		TheD/ThiD2 (Thiamine biosynthesis)	Cys451 active site/Cys111	
		Tpx (Thiol peroxidase)	Cys60 active site/Cys94 resolving	
		Mpx (Mycothiol peroxidase)	Cys36 active site	
		MsrA (Met-SO reductase)	Cys91 conserved	
		HmuO (Heme oxygenase)	Cys165	
		RpsC/F/M, RplM (ribosomal proteins)	Cys153/67/50/86	
		Tuf (translation elongation factor)	Cys277 conserved	
		PheT (Phe-tRNA synthetase)	Cys89 tRNA binding	

### The regulatory potential of protein *S*-glutathionylation in gram-negative bacteria

The role of protein *S*-glutathionylation for redox control has been studied in few Gram-negative bacteria, including *E. coli*, *S*. Typhimurium, *Neisseria meningitidis*, *Pseudoalteromonas haloplanktis*, and *Synechocystis* sp. PCC6803 (Table 3). In *E. coli*, the peroxide-sensing regulator OxyR is activated by *S*-glutathionylation at its redox-sensing Cys199 *in vitro* (Kim et al., 2002). In addition, the activities of glyceraldehyde-3-phosphate dehydrogenase, methionine synthase and the PAPS reductase are inhibited by *S*-glutathionylation in *E. coli* (Lillig et al., 2003; Hondorp and Matthews, 2004; Brandes et al., 2009). A recent study provides a model for the *S*-glutathionylation of the conserved active site Cys in GapDH and explains the reactivity of the active site toward H_2_O_2_ (Peralta et al., 2015). Reaction of the active site Cys with H_2_O_2_ is catalyzed by a mechanism which stabilizes the transition state and promotes leaving group departure by providing a proton relay. This model suggests the conserved redox-regulation of GapDH by *S*-thiolation of its active site Cys across all domains of life.

In *Neisseria meningitidis*, an esterase EstD acts together with the GSH-dependent alcohol dehydrogenase AdhC in formaldehyde detoxification. EstD is inactivated via *S*-glutathionylation at its conserved Cys54 by its substrate *S*-formyl-GSH during formaldehyde detoxification *in vitro* (Chen et al., 2013). In the psychrophilic bacterium *Pseudoalteromonas haloplanktis, S*-glutathionylation of the iron-superoxide dismutase PhSOD at the single Cys57 protected the enzyme from tyrosine nitration and peroxynitrite inactivation *in vitro* and *in vivo* (Castellano et al., 2008).

In *Synechocystis* sp. PCC6803, a MerA-like enzyme that functions in mercury and uranium reduction was shown to be redox-controlled by *S*-glutathionylation (Marteyn et al., 2013). MerA was *S*-glutathionylated at Cys78 that is required for mercury reduction resulting in inhibition of MerA activity. MerA redox regulation and reactivation required reduction by glutaredoxin-1 (Grx1). The active site Cys31 and Cys86 of Grx-1 operate in MerA interactions and both Cys are required for MerA reactivation. Furthermore, *S*-glutathionylation was shown to control the activity of the transcription factor AbrB2 in *Synechocystis* sp. PCC6803 (Cassier-Chauvat et al., 2014). AbrB2 is a repressor of the hydrogenase-encoding *hoxEFUYH* operon and also down-regulates antioxidant genes, such as *cydAB* encoding the cytochrome bd-quinol oxidase and *norB* encoding the nitric oxide reductase. The production of hydrogen is thought to be an antioxidant mechanism to eliminate electrons for oxygen reduction and ROS generation. AbrB2 contains a conserved single cysteine that is essential for redox-regulation and oligomerisation of AbrB2 as shown in C34S mutants. *S*-glutathionylation of Cys34 affected the binding of AbrB2 to the *hox* promoter and the stability of AbrB2 *in vitro*. In conclusion, *S*-glutathionylation has been shown to function in the redox-control of two transcriptional regulators, OxyR and AbrB2 in Gram-negative bacteria *in vitro*. However, compared to the many targets for *S*-glutathionylation that have been studied in eukaryotic organisms, there is much to be discovered about the regulatory potential of *S*-glutathionylation in bacteria.

Protein *S*-glutathionylation is a reversible redox switch mechanism. The glutaredoxin (Grx)/GSH/GSH reductase (Gor) system catalyzes specific de-glutathionylation of *S*-glutathionylated proteins (Fernandes and Holmgren, 2004; Inaba, 2009). Grx were first discovered in *E. coli* (Holmgren, 1976) where they have important functions as electron donors for ribonucleotide reductase (RNR), adenosine-5′-phosphosulfate (APS) reductase, 3′-phosphoadenosine-5′-phosphosulfate (PAPS) reductase and arsenate reductases (Holmgren, 1981; Aslund et al., 1994). Grx are structurally classified into the classical di-thiol Grxs with a *CPTC* redox active site and the monothiol Grx containing a *CGPS* redox active site (Lillig et al., 2008). In *E. coli*, three di-thiol Grx proteins (Grx1, Grx2, and Grx3) and one monothiol protein (Grx4) have been characterized.

The de-glutathionylation by Grx enzymes involves thiol-disulfide exchange reactions with GSH via nucleophilic double displacement (ping–pong) mechanisms and occurs via mono- or di-thiol mechanisms. Most di-thiol Grx use monothiol mechanisms that take place in two steps: In the first step, the nucleophilic thiolate anion attacks the *S*-glutathionylated substrate protein, resulting in reduction of the mixed disulfide and the *S*-glutathionylated Grx (Grx-SSG) intermediate. This Grx-SSG intermediate is regenerated by GSH and Gor at expense of NADPH (Allen and Mieyal, 2012). The di-thiol mechanism involves a second active site Cys that forms an intramolecular disulfide to resolve the Grx-SSG intermediate that has been shown for some plant Grx enzymes (Zaffagnini et al., 2012b). However, this di-thiol mechanism of Grx is less efficient for protein de-glutathionylation and more involved in the reduction of intermolecular protein disulfides (Lillig et al., 2008; Allen and Mieyal, 2012). Thus far, the knowledge about Grx functions and substrates in most GSH-producing bacteria is scarce and remains an important subject for future studies.

### Biosynthesis and regulation of bacillithiol in gram-positive firmicutes bacteria

Bacillithiol (BSH) is composed of Cys-GlcN-malate and serves as major LMW thiol in many Firmicutes bacteria, including *Bacillus* and *Staphylococcus* species, *Deinococcus radiodurans*, and *Streptococcus agalactiae* (Newton et al., 2009) (Figure 4). The BSH biosynthesis pathway was first identified in *B. subtilis*. In the first step, the glycosyltransferase BshA couples UDP-GlcNAc to L-malic acid for generation GlcNAc-Mal (Ruane et al., 2008; Gaballa et al., 2010; Parsonage et al., 2010). The deacetylase BshB1 catalyzes deacetylation of GlcNAc-Mal to GlcN-Mal. The last step involves the putative cysteine ligase YllA (BshC) that presumably adds Cys to GlcN-Mal (Gaballa et al., 2010). BshB1 has a paralog BshB2 and both enzymes have deacetylase activity. The functional redundancy of BshB1 and BshB2 in *B. subtilis* suggests that BshB2 might function as BSH-*S*-conjugate amidase Bca in detoxification of RES similar to the MSH-*S*-conjugate amidase Mca (Parsonage et al., 2010). The functions of the BshB1/2 homologs of *B. anthracis* (BA1557 and BA3888) and *B. cereus* (BC1534 and BC3461) in the deacetylation of GlcNAc-Mal have been demonstrated *in vitro*. In addition, BA3888 was shown to function as BSH-*S*-conjugate amidase (Bca) (Fang et al., 2013). In contrast to BshA and BshB, the activity of the putative cysteine ligase BshC has never been demonstrated biochemically *in vitro*. The structure of BshC was resolved revealing a core Rossmann fold with connecting peptide motifs (CP1 and CP2) and an α-helical coiled-coil domain required for dimerization (Vanduinen et al., 2015). BshC was crystallized with citrate and glycerol in the canonical active site and ADP bound in a second binding pocket that is different from the ADP-binding pocket in the related MshC structure. The active sites are solvent exposed and open for possible interactions with a protein, substrate or cofactor that remain to be elucidated to understand the catalytic mechanism of BshC (Vanduinen et al., 2015).

The regulation of the BSH biosynthesis genes has been studied in *B. subtilis*. The *bshA* and *bshB1* genes belong to a large operon of seven genes including *mgsA* which encodes a methylglyoxal synthase. The *bshB2* and *bshC* genes are encoded by two different operons. The *bshA*, *bshB*, and *bshC* genes are induced under conditions of disulfide stress provoked by diamide or NaOCl and positively controlled by the disulfide stress regulator Spx (Chi et al., 2011; Rochat et al., 2012; Gaballa et al., 2013). Consistent with the Spx-dependent control of the BSH biosynthesis genes, lower BSH levels were detected in the *spx* mutant using thiol-metabolomics (Chi et al., 2011; Rochat et al., 2012; Gaballa et al., 2013). It is interesting to note, that the Trx pathway and BSH biosynthesis genes are both regulated by the major disulfide stress regulator Spx in *B. subtilis* (Zuber, 2004, 2009; Chi et al., 2011; Rochat et al., 2012; Gaballa et al., 2013).

### Functions of bacillithiol and BSH-dependent detoxification enzymes

BSH is predominantly present in its reduced form in the cytoplasm with BSH/BSSB ratios ranging from 100:1 to 400:1 in *B. subtilis* indicating the presence of an efficient bacillithiol disulfide reductase (Sharma et al., 2013). The FAD-dependent pyridine nucleotide disulfide oxidoreductase YpdA (IPR023856) was suggested to function as BSSB reductase because of its phylogenetic relationship to the BSH biosynthesis enzymes as revealed by a STRING search (Gaballa et al., 2010). However, the function of YpdA has not yet been demonstrated.

The standard thiol-redox potential of BSH was calculated as *E*^0′^(BSSB/BSH) = −221 mV which is higher than the GSH redox potential [*E*^0′^(GSSG/GSH) = −240 mV] (Sharma et al., 2013). The microscopic p*K*_a_ values of the thiol group of BSH were determined as p*K*_a_ = 7.97 when the amino group of the Cys is protonated and as p*K*_a_ = 9.55 in the presence of the deprotonated amino group of Cys (Sharma et al., 2013). Thus, the thiol group in BSH is more acidic compared to the thiol group in Cys suggesting an enhanced level and reactivity of the BSH thiolate anions to detoxify reactive species. The BSH concentrations in *B. subtilis* vary during the growth in LB medium and increase strongly during the stationary phase to 3.5–5.2 mM. In contrast, the cellular Cys concentration is kept at a relatively low level (0.13–0.28 mM). Thus, BSH concentrations are ~17-fold higher compared to the level of Cys (Sharma et al., 2013). Similar concentrations of BSH (2 mM) were measured in *Bacillus pumilus* during growth. In *B. pumilus*, BSH levels increased under peroxide stress to 6 mM which is caused by an increased *bshB* expression (Handtke et al., 2014). BSH levels are also two-fold increased under diamide and NaOCl stress in *B. subtilis* due to Spx-dependent induction of *bshA, bshB*, and *bshC* (Chi et al., 2013; Gaballa et al., 2013). In *S. aureus*, the BSH levels are lower (0.3–1 mM) in the different clinical isolates (COL, USA300, Mu50, or N315) and BSH levels are not up-regulated during the stationary phase (Posada et al., 2014).

**Figure 6:**
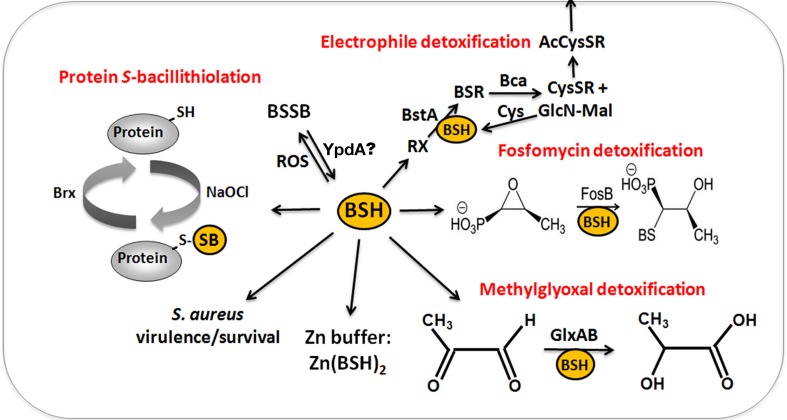
**The functions of bacillithiol (BSH) in *B. subtilis* and *S. aureus***. Bacillithiol functions in detoxification of ROS, RES, HOCl, and antibiotics (fosfomycin, rifampicin) in *B. subtilis* and *S. aureus*. BSH is oxidized by ROS to bacillithiol disulfide (BSSB). Electrophiles (RX) are conjugated to BSH by the BSH *S*-transferase BstA to form BS-electrophiles (BSR) which are cleaved by the BSH *S*-conjugate amidase Bca to CysSR and mercapturic acids (AcCySR) that are exported from the cell. BSH serves as a cofactor for the epoxide hydrolase FosB which adds BSH to fosfomycin to open the ring structure for its detoxification. BSH functions in methylglyoxal detoxification as a cofactor for the glyoxalases I/II (GlxA and GlxB) in *B. subtilis*. GlxA converts BSH-hemithioacetal to *S*-lactoyl-BSH that is further converted by GlxB to D-lactate. BSH serves as Zn buffer under conditions of Zn excess in *B. subtilis*. In *S. aureus*, BSH is important under infection-related conditions and increased the survival of S. aureus in phagocytosis assays using murine macrophages. Under conditions of NaOCl stress, proteins are oxidized to mixed disulfides with BSH, termed as *S*-bacillithiolations which is reversed by bacilliredoxins.

The physiological functions of BSH were studied in *bsh* mutants of *B. subtilis* and *S. aureus* (Table 1). Phenotype analyses showed increased sensitivities of *bsh* mutants toward hypochlorite, diamide, methylglyoxal, ROS (paraquat, H_2_O_2_), osmotic, and acidic stress, alkylating agents and fosfomycin in *B. subtilis* (Gaballa et al., 2010; Chi et al., 2011). The fosfomycin-sensitive phenotype of *bsh* mutants depends on the epoxide hydrolase FosB that requires BSH as a cofactor to open the ring structure for fosfomycin detoxification (Lamers et al., 2012; Roberts et al., 2013; Thompson et al., 2013) (Figure 6). FosB shows a preference for BSH as thiol cofactor and does only work poorly with Cys. The biochemical activity has been demonstrated for various *Bacillus* and *Staphylococcus* FosB homologs (Lamers et al., 2012; Roberts et al., 2013; Thompson et al., 2013). In *B. subtilis* and *S. aureus*, both FosB and BSH confer resistance to fosfomycin treatment in survival assays *in vivo* (Gaballa et al., 2010; Thompson et al., 2014). Co-crystallization of *S. aureus* FosB with L-Cys or BSH revealed a mixed disulfide at the active site Cys9 of FosB which is unique in FosB from *S. aureus* (Thompson et al., 2014).

Reactive electrophiles, such as monobromobimane are detoxified by direct conjugation to BSH or by conjugation reactions catalyzed by BSH *S*-transferases. BSH functions as a cofactor for DinB-family *S*-transferases that are widely distributed among GSH-, BSH-, and MSH-producing bacteria (Newton et al., 2011; Perera et al., 2014). The *B. subtilis* DinB-family YfiT protein was active as *S*-transferase with BSH to conjugate monochlorobimane, but inactive with MSH or GSH (Newton et al., 2011). The *yfiT* gene is flanked by *yfiS* and *yfiU* encoding putative efflux transporters for mercapturic acids produced during electrophile detoxification. The YfiT-homolog of *S. aureus* BstA catalyzed the conjugation of BSH to monochlorobimane, 1-chloro-2,4-dinitrobenzene and cerulenin, while rifampicin was BstA-independently conjugated to BSH (Perera et al., 2014).

BSH is involved in methylglyoxal detoxification and functions as a cofactor for BSH-dependent glyoxalases in *B. subtilis* (Chandrangsu et al., 2014). Methylglyoxal rapidly depletes BSH leading to BSH-hemithioacetal formation that is converted to *S*-lactoyl BSH by the glyoxalase-I (GlxA). The glyoxalase-II (GlxB) catalyzes conversion of *S*-lactoyl-BSH to lactate (Figure 6). Phenotype studies further indicated that BSH can detoxify heavy metal ions, such as tellurite and selenite in *B. subtilis* (Helmann, 2011). In addition, BSH functions as Zn buffer in metal ion homeostasis (Ma et al., 2014). The Cys thiol and carboxylate moieties of BSH can bind and store Zn(II) as BSH_2_:Zn complex under conditions of Zn(II) stress (Ma et al., 2014). BSH binding to Zn(II) occurred at much higher affinity compared to GSH. Mutants lacking BSH are more sensitive to Zn(II) stress and induced the Zn efflux CadA system at lower Zn levels compared to the wild type. BSH also protected against Zn(II) toxicity in cells lacking Zn efflux pumps. In addition, Zn efflux is elevated under conditions of diamide stress when the pool of reduced BSH is depleted. These results establish a new role of BSH as buffer for the labile Zn pool that are likely important for related pathogens under infection conditions.

In conclusion, functional analyses of *bsh* mutants established important roles of BSH as GSH surrogate in Firmicutes bacteria, including similar detoxification functions and BSH-dependent enzymes, such as DinB-family *S*-transferases and glyoxalases that are widely conserved across bacteria. However, the conserved role of FosB as BSH-dependent fosfomycin hydrolase and the function of BSH as Zn buffer have been described only in BSH-producing bacteria.

### Functions of bacillithiol in the virulence of *Staphylococcus aureus*

Phenotype analyses of *S. aureus bsh* mutants were conducted for different clinical isolates of methicillin-resistant *S. aureus* strains (MRSA) that revealed a role of BSH for stress resistance and under infection conditions (Pöther et al., 2013; Posada et al., 2014) (Table 2). In survival assays, *S. aureus* USA300 LAC transposon *bsh* mutants were more sensitive to alkylating agents (iodoacetamide and CDNB), methylglyoxal, peroxide and superoxide stress, diamide, fosfomycin, cerulenin, rifamycin and metals ions, like copper and cadmium (Rajkarnikar et al., 2013). In *S. aureus* COL and USA300 backgrounds, *bshA* and *fosB* mutants with clean deletions showed increased sensitivities to fosfomycin, diamide and H_2_O_2_ and the levels of NADPH and BSH were decreased in *fosB* mutants suggesting a function of FosB as *S*-transferase in the oxidative stress resistance (Posada et al., 2014). The *S. aureus* COL and USA300 *bshA* mutants showed a decreased survival in human whole-blood survival assays (Posada et al., 2014). Microarray analyses of the *bshA* mutant further revealed that staphyloxanthin biosynthetic genes are induced while the level of staphyloxanthin was strongly decreased in the *S. aureus bshA* mutant. Interestingly, the widely used strains of the *S. aureus* NCTC8325 lineage including SH1000 harbor natural *yllA* (*bshC*) null mutations that are caused by a 8 bp duplication in the *bshC* gene and these strains do not produce BSH (Gaballa et al., 2010; Newton et al., 2012; Posada et al., 2014). In contrast, *S. aureus* Newman encodes a functional *bshC* gene and produces BSH as revealed by thiol metabolomics (Newton et al., 2012; Pöther et al., 2013). BSH biosynthesis in *S. aureus* SH1000 could be restored by plasmid-encoded expression of the *bshC* gene (Pöther et al., 2013; Posada et al., 2014). In phagocytosis assays using murine macrophages and human epithelial cell lines the survival of the SH1000 strain was decreased compared to the *bshC* complemented *S. aureus* strain (Pöther et al., 2013; Posada et al., 2014). Thus, BSH is involved in the defense against the host-immune system and contributes to pathogen fitness in *S. aureus* clinical MRSA isolates under infection-related conditions. It will be exciting to unravel the regulatory mechanisms that contribute to virulence control by BSH in *S. aureus*.

### The role of protein *S*-bacillithiolation in gram-positive firmicutes bacteria

**Figure 7:**
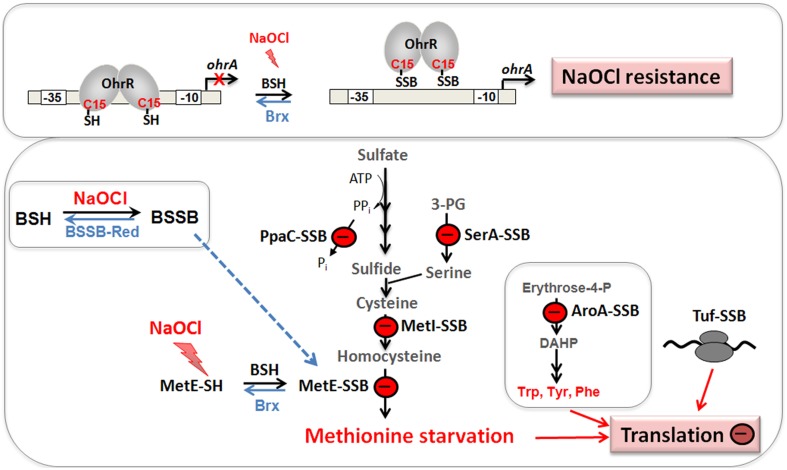
**Physiological roles of *S*-bacillithiolations in *B. subtilis* and other Firmicutes**. NaOCl leads to *S*-bacillithiolation of OhrR, MetE, YxjG, PpaC, SerA, AroA, GuaB, YumC, TufA, and YphP in *B. subtilis* (Chi et al., 2011). *S*-bacillithiolation of OhrR inactivates the repressor and causes induction of the OhrA peroxiredoxin that confers NaOCl resistance. *S*-bacillithiolation of the methionine synthase MetE at its active site Cys730 and other enzymes of the Cys and Met biosynthesis pathway (YxjG, PpaC, SerA, MetI) leads to methionine auxotrophy (Chi et al., 2011, 2013). In addition, other amino acids biosynthesis enzymes, translation factors and ribosomal proteins are *S*-bacillithiolated in Firmicutes bacteria. Thus, we hypothesize that *S*-bacillithiolation leads to a transient translation stop during the time of NaOCl detoxification to prevent further protein damage. NaOCl stress causes oxidation of BSH to BSSB and a two-fold decreased BSH/BSSB redox ratio that possibly contributes to *S*-bacillithiolation. The reduction of MetE-SSB and OhrR-SSB is catalyzed by bacilliredoxins (BrxA/B) in *B. subtilis*.

Protein *S*-bacillithiolation was recently discovered as a widespread thiol protection and redox-regulatory mechanism in different Firmicutes bacteria (Chi et al., 2011, 2013) (Figure 7). *S*-bacillithiolation functions as a redox-switch mechanism to control the activity of redox-sensing transcription factors and metabolic enzymes, including OhrR and MetE (Lee et al., 2007; Chi et al., 2011) (Table 3). *S*-bacillithiolation of the OhrR repressor occurs at its lone Cys15 residue leading to inactivation of OhrR and expression of the thiol-dependent OhrA peroxiredoxin for detoxification of organic hydroperoxides and NaOCl (Fuangthong et al., 2001; Chi et al., 2011). *S*-bacillithiolation is also widespread among other Firmicutes with eight common and 29 unique *S*-bacillithiolated proteins identified in *B. subtilis, Bacillus amyloliquefaciens, Bacillus pumilus, B. megaterium*, and *Staphylococcus carnosus* (Chi et al., 2011, 2013). The *S*-bacillithiolome contains mainly biosynthetic enzymes for amino acids (methionine, cysteine, branched chain and aromatic amino acids), cofactors (thiamine), nucleotides (GTP), as well as translation factors, chaperones, redox, and antioxidant proteins. Among the most conserved protein-SSB were abundant and essential proteins like TufA, MetE, GuaB that are targets for *S*-thiolation also in MSH-producing bacteria (Chi et al., 2014).

The methionine synthase MetE is the most abundant *S*-bacillithiolated protein in *Bacillus* species after NaOCl exposure. *S*-bacillithiolation of MetE occurs at its Zn-binding active site Cys730 and at the non-essential surface-exposed Cys719, leading to methionine starvation in NaOCl-treated cells (Chi et al., 2011). Similarly, methionine auxotrophy is caused by *S*-glutathionylation of MetE in *E. coli* after diamide stress (Hondorp and Matthews, 2004). The active site Zn center of MetE is also *S*-mycothiolated in *C. glutamicum* (Chi et al., 2014). Since formyl methionine is required for initiation of translation, MetE inactivation could stop translation during the time of hypochlorite detoxification. This translation arrest caused by *S*-bacillithiolation is supported by the strong repression of the stringent response RelA regulon under NaOCl stress, which includes genes for ribosomal proteins and translation factors (Chi et al., 2011).

Our studies revealed that *S*-bacillithiolations were observed under diamide and NaOCl stress, but not under control conditions. This confirms previous results about the mechanisms of *S*-glutathionylations which requires activation of protein thiols by ROS. *S*-glutathionylation can be caused via thiol-disulfide exchange with GSSG and by activation of thiols to sulfenic acid, sulfenylamides, thiyl radicals, thiosulfinate or *S*-nitrosyl intermediates (Gallogly and Mieyal, 2007; Mieyal et al., 2008; Allen and Mieyal, 2012; Mieyal and Chock, 2012). Hypochlorite leads to chlorination of the thiol group to form sulfenylchloride that is unstable and rapidly reacts further to form mixed BSH protein disulfides (Hawkins et al., 2003; Davies, 2011). The increased BSSB level under NaOCl-stress might also contribute to *S*-bacillithiolation via thiol-disulfide exchange.

**Figure 8:**
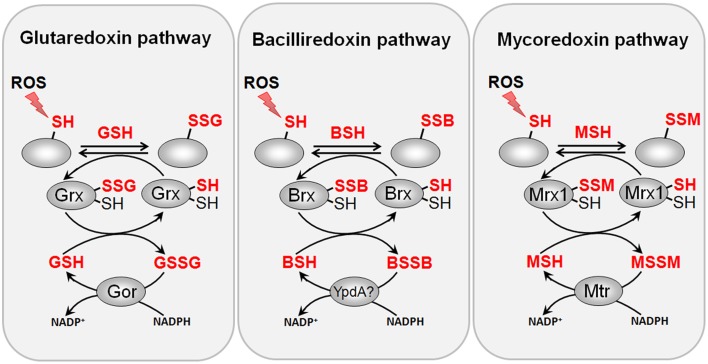
**Reduction of protein *S*-glutathionylations, *S*-bacillithiolations and *S*-mycothiolations by glutaredoxin, bacilliredoxin and mycoredoxin pathways**. The *S*-glutathionylated proteins are reduced by glutaredoxins (Grx) leading to a Grx-SSG intermediate that is reduced by GSH leading to GSSG which is recycled back to GSH by the NADPH-dependent GSSG reductase (Gor). Analogous bacilliredoxin and mycoredoxin pathways have been characterized in BSH- and MSH-utilizing Gram-positive bacteria. The *S*-bacillithiolated proteins are reduced by bacilliredoxins (Brx) leading to Brx-SSB formation. Brx-SSB is reduced by BSH with the generation of BSSB that likely requires the NADPH-dependent BSSB reductase YpdA for regeneration of BSH. In Actinomycetes, mycoredoxin1 catalyzes reduction of *S*-mycothiolated proteins leading to Mrx1-SSM generation that is recycled by MSH and the NADPH-dependent MSSM reductase Mtr.

Among the *S*-bacillithiolated proteins, the thioredoxin-like proteins YtxJ, YphP, and YqiW were identified in *B. subtilis* and *Staphylococcus* that occur only in BSH-producing bacteria (Chi et al., 2013). These Trx-like enzymes were suggested to function as bacilliredoxins (Brx) in the de-bacillithiolation process. YtxJ could functions as monothiol Brx and contains a single Cys in the conserved TCPIS motif. YphP (BrxA) and YqiW (BrxB) are paralogs of the uncharacterized DUF1094 family (53% identity) with unusual CGC active sites (Gaballa et al., 2010). YphP has also weak thiol-disulfide isomerase activity and a relatively high standard redox potential of *E*^0′^ = −130 mV (Derewenda et al., 2009). It was demonstrated that BrxA and BrxB function in the reduction of the *S*-bacillithiolated substrates MetE and OhrR *in vitro* (Gaballa et al., 2014) (Figure 8). The BrxBCxA resolving Cys mutant protein was able to reduce *S*-bacillithiolated OhrR to restore the DNA-binding activity of OhrR. However, the BrxBCxA mutant was unable to reduce *S*-cysteinylated OhrR. These results provide first evidence for the function of glutaredoxin-like enzymes in BSH-producing bacteria. However, phenotype analyses revealed that both, BrxA and BrxB are not essential and rather dispensable for oxidative stress resistance under conditions of *S*-bacillithiolations in *B. subtilis* (Gaballa et al., 2014). Thus, the bacilliredoxin pathway is redundant with other thiol-disulfide oxidoreductases or the thioredoxin pathway *in vivo* for reduction of BSH mixed disulfides. In conclusion, the redox regulation of enzymes and transcription regulators by *S*-bacillithiolation and bacilliredoxins has been studied in detail in the model bacterium *B. subtilis*. Future studies should be directed to elucidate if *S*-bacillithiolation and bacilliredoxins control virulence functions and pathogen fitness in the major pathogen *S. aureus*.

### Biosynthesis and regulation of mycothiol in actinomycetes

Mycothiol (MSH) is composed of N-Acetyl-Cys-GlcN-myoinositol (Figure 4) and is present in high-GC Gram-positive Actinomycetes, such as Streptomycetes, Mycobacteria and Corynebacteria (Jothivasan and Hamilton, 2008; Newton et al., 2008). The biosynthesis of MSH proceeds from myo-inositol-1-phosphate, UDP-GlcNAc and cysteine and occurs in five steps (Jothivasan and Hamilton, 2008; Newton et al., 2008). The glycosyltransferase MshA conjugates *myo*-inositol-1-P to UDP-GlcNAc and produces GlcNAc-Ins-P. Dephosphorylation of GlcNAc-Ins-P by the phosphatase MshA2 generates GlcNAc-Ins which is the substrate for the deacetylase MshB. The MshB enzyme is homologous to the MSH S-conjugate amidase (Mca), and has both deacetylase and amidase activities. The cysteine ligase MshC adds Cys to GlcN-Ins to generate Cys-GlcN-Ins. The final acetylation of the Cys is catalyzed by the acetyltransferase MshD to produce MSH (Jothivasan and Hamilton, 2008; Newton et al., 2008). The structure of MSH is similar to that of BSH and the glycosyltransferase BshA and deacetylase BshB of *B. subtilis* are homologs of the MshA and MshB enzymes of Mycobacteria.

MSH biosynthesis enzymes in *Streptomycetes* are redox-controlled under diamide stress by the disulfide stress specific σ^R^ ECF sigma factor/RsrA anti sigma factor system (Kim et al., 2012). σ^R^ is sequestered by its redox-sensitive anti sigma factor RsrA in non-stressed cells. RsrA is oxidized at redox-sensing Cys residues in the Zn-binding site under disulfide stress that leads to relief of σ^R^. Free σ^R^ transcribes genes required to maintain the thiol-redox homeostasis, including the genes for TrxAB and MSH biosynthesis, such as *mshA, mshB*, *mshC, mshD*, *mca* (Bae et al., 2004; Newton and Fahey, 2008; Park and Roe, 2008). In *C. glutamicum*, the homologous ECF sigma factor σ^H^/RshA system controls the disulfide stress response genes for the Trx/TrxR system (*trxB, trxB1, trxC*) and for MSH biosynthesis and recycling (*mshC, mca, mtr*) (Ehira et al., 2009; Busche et al., 2012). The regulation of the Trx and MSH pathways by σ^R^/RsrA or σ^H^/RshA is conserved among Actinomycetes (Park and Roe, 2008; Antelmann and Helmann, 2011; Kim et al., 2012). Thus, it is common in Gram-positive bacteria that the genes for BSH and MSH biosynthesis pathways are under redox-control of the major disulfide stress regulators, Spx in Firmicutes bacteria and RsrA/RshA in Actinomycetes, respectively.

### Functions of mycothiol and MSH-dependent enzymes in actinomycetes

**Figure 9:**
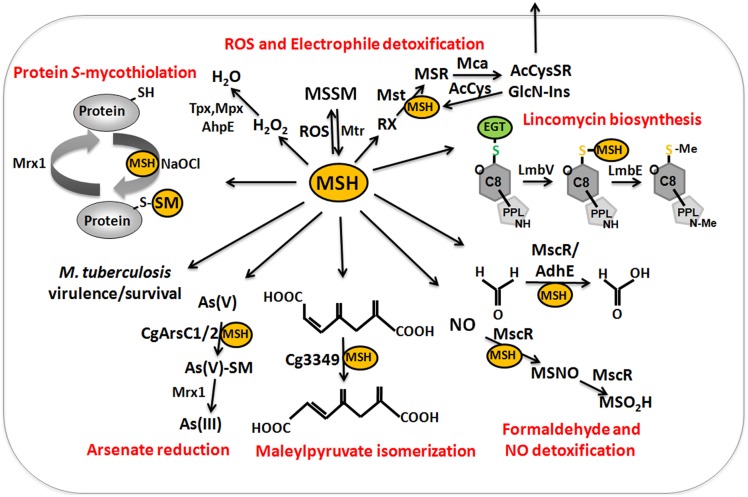
**The functions of mycothiol (MSH) in Mycobacteria and Corynebacteria**. Mycothiol (MSH) is oxidized by ROS to mycothiol disulfide (MSSM). MSSM is reduced back to MSH by the mycothiol disulfide reductase Mtr on expense of NADPH. MSH-dependent peroxidases, such as Mpx, Tpx, and AhpE function in peroxide detoxification. Electrophiles (RX) are conjugated to MSH by the MSH *S*-transferase Mst to form MS-electrophiles (MSR) which are cleaved by the MSH *S*-conjugate amidase Mca to mercapturic acids (AcCySR) that are exported from the cell. The Mca-homologs LmbT, LmbV, and LmbE function also in the assembly and biosynthesis of the sulfur-containing lincosamide antibiotic lincomycin in *Streptomyces lincolnensis* (Zhao et al., 2015). MSH serves as a cofactor for the alcohol dehydrogenase AdhE/MscR in Mycobacteria and Corynebacteria for detoxification of formaldehyde to formate and MSNO to MSO2H. MSH functions in detoxification of maleylpyruvate as a cofactor for maleylpyruvate isomerase in *C. glutamicum*. Arsenate reductases CgArsC1 and CgArsC2 conjugate MSH and arsenate As(V) to form As(V)-SM that is reduced to As(III) by Mrx1. In *M. tuberculosis*, MSH is important under infection conditions and for growth and survival. Under conditions of NaOCl stress, proteins are oxidized to mixed disulfides with MSH, termed as *S*-mycothiolations which is reversed by mycoredoxins.

MSH serves as the major thiol-redox buffer in Actinomycetes. MSH is oxidized to MSH disulfide (MSSM) under oxidative stress conditions. The mycothiol disulfide reductase Mtr maintains MSH in its reduced state at the expense of NADPH. MSH is involved in protection against oxidative and electrophile stress, alkylating agents, toxins, antibiotics (erythromycin, vancomycin, rifampin, azithromycin), heavy metal stress, aromatic compounds, ethanol and glyphosate in Streptomycetes, Mycobacteria and Corynebacteria (Buchmeier et al., 2003, 2006; Rawat et al., 2007; Newton et al., 2008; Liu et al., 2013) (Table 1). MSH is used as a cofactor for MSH-dependent enzymes during detoxification of toxins, electrophiles and antibiotics in Actinomycetes (Figure 9, Table 1). MSH forms conjugates with xenobiotics and antibiotics either spontaneously or by the DinB-family MSH *S*-transferases (Newton et al., 2011). The MSH *S*-transferase Mst of *M. smegmatis* was shown to catalyze the conjugation of monochlorobimane and DTNB to MSH but its natural substrate is not known (Newton et al., 2011). MSH-*S*-conjugates are rapidly cleaved by the MSH-*S*-conjugate amidase (Mca) to glucoseamine-myo-inositol (GlcN-Ins) and mercapturic acid derivatives (AcCysSR) that are excreted from the cell. Mca is the major detoxification enzyme for MSH *S*-conjugates with antibiotics, including cerulenin and rifamycin in *Mycobacteria* (Newton et al., 2008, 2011). Interestingly, MSH and the Mca-homologs LmbT, LmbV and LmbE play also a direct role in the biosynthesis of the sulfur-containing lincosamide antibiotic lincomycin in *Streptomyces lincolnensis* (Zhao et al., 2015). MSH functions as the sulfur donor for incorporation of the methylmercapto group into lincomycin after thiol exchange. In addition, ergothioneine (EGT), that is utilized as another thiol by Actinomycetes, acts as a carrier for the assembly of the N-methylated 4-propyl-L-proline (PPL) and lincosamide moieties to form lincomycin. EGT and MSH were shown to function in lincomycin biosynthesis through unusual *S*-glycosylations documenting a first biochemical role of LMW thiols in bacteria. Since the biosynthetic pathways for many sulfur-containing natural compounds include Mca homologs, the involvement of LMW thiols in natural product biosynthesis might be a common mechanism (Zhao et al., 2015).

MSH functions as a cofactor for many redox enzymes that are involved in the detoxification of peroxides, electrophiles (formaldehyde), NO, aromatic compounds (maleylpyruvate) and arsenate (Fahey, 2013) (Table 1). There is evidence for a MSH-peroxidase Mpx involved in peroxide detoxification that was identified as *S*-mycothiolated Gpx-homolog under oxidative stress in *C. glutamicum* (Chi et al., 2014). The MSH-dependent alcohol dehydrogenase MscR (MSNO reductase/formaldehyde dehydrogenase) catalyzes the detoxification of formaldehyde and *S*-nitrosyl-mycothiol (MSNO) (Newton et al., 2008). MSH reacts with formaldehyde to MS-CH_2_OH that is converted to formate by MscR. MscR also converts MSNO to MSH sulfinamide (MSONH_2_). In *C. glutamicum*, a similar MSH-dependent pathway for formaldehyde oxidation by the MSH-dependent formaldehyde dehydrogenase AdhE/FadH has been characterized (Lessmeier et al., 2013; Witthoff et al., 2013). In *C. glutamicum*, MSH is further involved in degradation of aromatic compounds, including gentisate, 3-hydroxybenzoate, maleylpyruvate, resorcinol, and naphthalene and *msh* mutants were unable to grow on these substrates (Liu et al., 2013). MSH functions as a cofactor for the maleylpyruvate isomerase in the gentisate ring-cleavage pathway to catalyze the isomerization of maleylpyruvate to fumaryl pyruvate in *C. glutamicum* (Feng et al., 2006). Similarly, MSH was suggested as a cofactor for enzymes of the naphthalene and resorcinol degradation pathway (Liu et al., 2013).

MSH confers resistance to metal ions, such as Cr(VI), Zn(II), Cd(II), Co(II), and Mn(II) in *C. glutamicum* (Liu et al., 2013). The detoxification of arsenate [As-(V)] to arsenite [As(III)] depends on the MSH-dependent arsenate reductases ArsC1/C2 (Ordonez et al., 2009). ArsC1/C2 function similar to *S*-transferases in arsenate detoxification by formation of an arseno-MSH conjugate that requires the mycoredoxin-1/MSH/Mtr electron pathway for reduction. In contrast, another arsenate reductase Cg_ArsC1′ detoxifies arsenate with electrons from the Trx pathway (Villadangos et al., 2011).

MSH enhanced also the robustness of *C. glutamicum* during industrial production of glutamate and L-lysine (Liu et al., 2014). The overexpression of *mshA* resulted in increased MSH biosynthesis and higher resistance of *C. glutamicum* to peroxides, methylglyoxal, antibiotics (erythromycin and streptomycin), metal ions, organic acids, furfural and ethanol (Liu et al., 2014). Thus, the increased biosynthesis of LMW thiol redox buffers, as shown for GSH in *C. acetobutylicum* and MSH in *C. glutamicum*, might be a promising strategy to engineer robust industrial production strains.

In *Mycobacterium tuberculosis*, MSH is essential for growth and survival of *M. tuberculosis* under infection conditions (Sareen et al., 2003; Sassetti and Rubin, 2003). In addition, MSH is required to activate the antituberculosis prodrug isoniazid and hence *M. tuberculosis mshA* mutants are resistant to isoniazid (Buchmeier et al., 2003). Tuberculosis (TB) causes still nearly 2 million death each year and multiple and extensive drug resistant strains occur that require new targets for antituberculosis drugs. Thus, inhibitors of MSH biosynthesis enzymes are promising candidates for antituberculosis drug developments. Several MSH biosynthesis inhibitors have been applied that target the MSH-*S*-conjugate amidase Mca, the deacetylase MshB, the cysteine ligase MshC and the MSSM reductase Mtr that are attractive antituberculosis drug targets (Nilewar and Kathiravan, 2014).

### The role of protein *S*-mycothiolation in gram-positive actinomycetes

**Figure 10:**
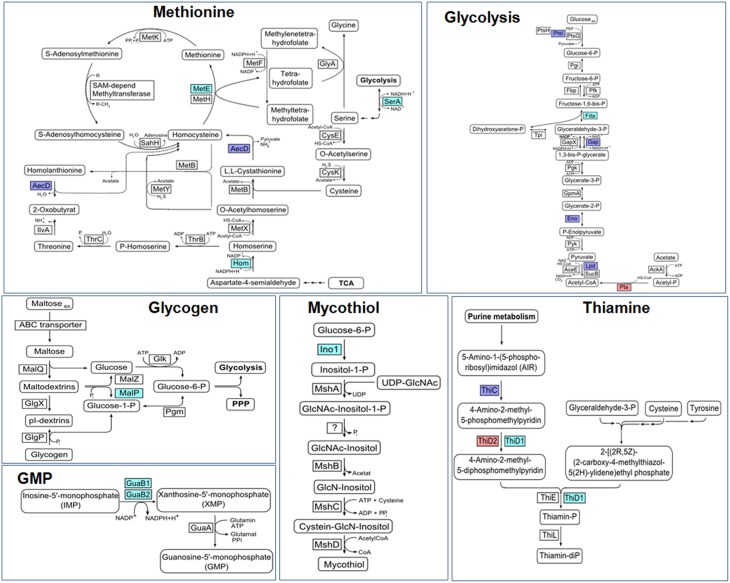
**Physiological roles of *S*-mycothiolations in *Corynebacterium glutamicum***. The metabolic pathways for glycolysis, biosynthesis of methionine, thiamine, GMP, MSH, and glycogen metabolism are shown including identified *S*-mycothiolated proteins. The identified *S*-mycothiolated or oxidized proteins are labeled with colors (*S*-mycothiolated proteins are red; reversibly oxidized proteins are magenta; both reversibly oxidized and *S*-mycothiolated are blue). The selected *S*-mycothiolated metabolic enzymes include MetE, SerA, Hom (Met biosynthesis); Fba, Pta (glycolysis); MalP (glycogen utilization); Ino-1 (MSH biosynthesis); ThiD1, ThiD2 (thiamine biosynthesis); GuaB1, GuaB2 (GMP biosynthesis). Further proteins with Cys-SSM sites are involved in translation (Tuf, PheT, RpsC, RpsF, RpsM, RplM) and antioxidant functions (Tpx, Bcp, MsrA) that are not shown here. The figure is adapted from (Chi et al., 2014).

Protein *S*-mycothiolation was first studied in *C. glutamicum* and 25 *S*-mycothiolated proteins could be identified under NaOCl stress by mass spectrometry (Chi et al., 2014) (Table 3). The thiol-peroxidase Tpx and the putative MSH peroxidase Mpx were *S*-mycothiolated under control and NaOCl stress conditions at their active site Cys residues. The fragment ion spectra of the *S*-mycothiolated Cys-peptides are characterized by diagnostic myoinositol-loss precursor ions (−180 Da) that serve as markers for identification. The 25 *S*-mycothiolated proteins overlap with 16 NaOCl-sensitive proteins identified in the fluorescent-label thiol-redox proteome. These include Tuf, GuaB1, GuaB2, SerA, and MetE as conserved abundant targets for *S*-thiolations across Gram-positive bacteria (Chi et al., 2013). The *S*-mycothiolated proteins are involved in the metabolism of carbohydrates, such as glycolysis (Fba, Pta, XylB), glycogen and maltodextrin degradation (MalP) and several biosynthesis pathways for serine, cysteine, methionine (SerA, Hom, MetE), nucleotides and thiamine (GuaB1, GuaB2, PurL, NadC, ThiD1, and ThiD2) and myo-inositol-1-phosphate (Ino-1 or Cg3323) (Figure 10). Further protein-SSM function in peroxide detoxification (Tpx, Gpx), methionine sulfoxide reduction (MsrA), heme degradation for iron mobilization (HmuO) and protein translation (RpsF, RpsC, RpsM, RplM, Tuf). The glycogen phosphorylase MalP is one of the most abundantly *S*-mycothiolated proteins in NaOCl-treated cells (Chi et al., 2014). *S*-mycothiolation of MalP is important for oxidative stress resistance in *C. glutamicum* since the *malP* deletion mutant is NaOCl-sensitive in growth assays. MalP functions in glycogen degradation during the stationary phase. *S*-mycothiolation of MalP may prevent glycogen degradation under NaOCl stress since the glycogen content remained stable despite a strongly decreased glucose uptake rate.

The mycoredoxin-1 (Mrx1) has been characterized as glutaredoxin-homolog of Actinomycetes in reduction of MSH mixed disulfides (Van Laer et al., 2012) (Figure 8). Mrx-1 has a typical Trx-like fold with a CGYC motif and a *cis*-Pro57 in a groove that presumable binds MSH. The redox potential of Mrx-1 was calculated as *E*^0′^ = −218 mV and the pK_a_ of the active site Cys17 was 5.1–5.6. Mrx-1 catalyzed de-mycothiolation in a hydroxyethyl disulfide (HED) assay and is coupled to the MSH/Mtr/NADPH pathway. Mrx-1 operates via a monothiol reaction mechanism in the de-mycothiolation reaction analogous to most glutaredoxins that are involved in de-glutathionylation. The first Mrx1 substrate was identified as the thiol-peroxidase Tpx that was *S*-mycothiolated at its active site Cys60 and resolving site Cys94 in *C. glutamicum in vivo* under hypochlorite stress (Chi et al., 2014). Tpx showed NADPH-linked peroxidase activity and reduced H_2_O_2_ in a Trx/TrxR-coupled electron assay. *S*-mycothiolation of Tpx inhibits the peroxidase activity which was restored after reduction by the Mrx1/MSH/Mtr pathway. Thus, *S*-mycothiolation controls Tpx activity and protects the peroxidatic Cys against overoxidation. In *M. tuberculosis*, Mrx1 has been shown to reduce the one-Cys peroxiredoxin AhpE (Hugo et al., 2014). AhpE is a membrane-associated peroxidase that detoxifies peroxinitrite and fatty acid hydroperoxides as preferred substrates (Hugo et al., 2009; Reyes et al., 2011). AhpE is oxidized by peroxides to form a sulfenic acid intermediate (AhpE-SOH) that can be reduced directly by Mrx1. Alternatively, AhpE-SOH can react with MSH to *S*-mycothiolated AhpE-SSM which is reduced by the Mrx1/MSH/Mtr electron pathway (Hugo et al., 2014). The direct AhpE-SOH reduction may occur in the membrane when MSH is not available and the formation of AhpE-SSM and subsequent Mrx1-reduction was suggested to predominate in the cytosol. Interestingly, the reducing mechanism of AhpE-SSM is similar to the detoxification of arsenate by CgArsC1 and CgArsC2. Arsenate reacts with MSH to an arseno-(V)-MSH complex that is reduced by Mrx1 releasing As(III) and Mrx1-SSM that is recycled by the MSH/Mtr/NADPH electron pathway (Ordonez et al., 2009; Villadangos et al., 2011). It remains to be shown if AhpE is mycothiolated under oxidative stress in *M. tuberculosis* cells *in vivo*. These results show that Mrx1 functions as glutaredoxin homolog in *C. glutamicum* and *M. tuberculosis* in the reduction of *S*-mycothiolated peroxiredoxins (Tpx and AhpE), when coupled to the MSH/Mtr/NADPH electron pathway and as electron donor for arsenate reductase in arsenate detoxification.

Recently, Mrx1 has been coupled to redox sensitive GFP (roGFP2) to construct a new genetically encoded biosensor for dynamic measurements of the MSH redox potential in different *M. tuberculosis* strains (Bhaskar et al., 2014). This study revealed phenotypic redox heterogeneity of *E*^0′^(MSSM/MSH) within Mycobacteria inside infected macrophages that are caused by sub-vacuolar compartments. Those sub-populations with higher *E*^0′^(MSSM/MSH) were more susceptible to clinical relevant antibiotics whereas populations with lower MSH redox potentials were resistant to antibiotics. The results further show that several anti-TB drugs induce oxidative stress in *M. tuberculosis* during infections. In conclusion, this Mrx1-roGFP2 biosensor is a promising tool to study MSH redox potential changes of *M. tuberculosis* under infections and antibiotic treatments. This is the first example for a genetically encoded redox biosensor that measures dynamic changes of the mycothiol redox potential in bacteria. Future studies should be directed to apply similar biosensors in other pathogenic bacteria to study the dynamics of redox potential changes during infections.

## Conclusion and perspectives for future research

In this review, we provide an overview about the biosynthesis pathways and functions of the bacterial redox buffers glutathione, bacillithiol and mycothiol and their regulatory roles for protein *S*-thiolations. Bacterial redox buffers maintain the reduced state of the cytoplasm and function as cofactors of conserved enzymes for detoxification of ROS, RES, chlorines, antibiotics and xenobiotics. These thiol-dependent enzymes include NADPH-dependent disulfide reductases (Gor, Mtr, YpdA) and related glutaredoxin-like enzymes (Grx, Mrx, Brx), DinB-family *S*-transferases (Gst, Mst, BstA), *S*-conjugate amidases (Mca, Bca) and glyoxalases (GloAB, GlxAB). However, some detoxification enzymes still need to be characterized in BSH-utilizing bacteria, including the BSH-dependent formaldehyde reductase (AdhA), the putative BSH peroxidase (Bpx) or thiol-dependent dioxygenases (MhqA, MhqE and MhqO) (Antelmann et al., 2008). The discovery of the biochemical functions of MSH, EGT and *S*-transferases in the lincomycin antibiotic biosynthesis opens perspectives to characterize the roles of thiol-redox buffers in the biosynthesis of sulfur-containing co-factors, natural compounds and antibiotics in other bacteria.

The structures of BSH and MSH are similar and the BSH biosynthesis enzymes BshA, BshB and BshC are homologous to the MSH biosynthesis enzymes MshA, MshB, and MshC. However, the crystal structure of BshC has revealed significant differences compared to MshC which requires further studies to understand the still unknown cysteine ligation mechanism of BshC (Vanduinen et al., 2015). It is further interesting, that the levels of BSH and MSH vary strongly between Firmicutes and Actinomycetes and also during growth and stress conditions. While Mycobacteria produce up to 20 mM MSH, the levels of BSH are much lower reaching 1–6 mM in Firmicutes bacteria. The differences in BSH and MSH levels during growth and under stress can be explained by the redox control of the BSH and MSH biosynthesis enzymes by the major thiol-based redox sensors (Spx and RsrA/RshA), presumably to enhance the redox buffer capacity under certain conditions to keep the reduced state of the cytoplasm. In contrast, redox regulation of GSH biosynthesis genes has not been shown. However, the pathogen *L. monocytogenes* is able to synthesize GSH and to import host-derived GSH as adaptation strategy under infection conditions (Reniere et al., 2015). Importantly, synthesized and host-derived GSH both contribute to virulence factor regulation in *L. monocytogenes*, while GSH-import was required for full virulence in *S. pneumoniae* (Potter et al., 2012; Reniere et al., 2015). Overall, the roles of GSH, BSH and MSH for virulence and pathogen fitness have been shown for many important human pathogens, including *L. monocytogenes*, *S. pneumoniae, S*. Typhimurium, *S. aureus*, and *M. tuberculosis*. Future studies in the field of infection biology should be directed to understand the molecular mechanisms of virulence factor regulation by thiol-redox buffers that might involve also protein *S*-thiolation mechanisms. The GSH, BSH and MSH biosynthesis enzymes, GSH uptake systems as well as *S*-thiolated proteins could be promising drug targets for the development of novel anti-infectives against emerging drug resistant strains of *S. pneumoniae*, *S. aureus* and *M. tuberculosis*. Thus, the large scale identification and quantification of *S*-thiolated proteins in pathogens is an important topic for future research.

Advances in mass spectrometry and chemical probe design have facilitated the development of more sensitive redox proteomics methods, such as the NEM-biotin switch assay or the Gsp-biotin assay to study targets for protein *S*-glutathionylation at a global scale (Lind et al., 2002; Kehr et al., 2011; Lin et al., 2015). In addition, numerous BSH- and MSH-mixed protein disulfides have been identified recently under disulfide stress conditions, such as NaOCl and diamide. However, more quantitative MS-based redox proteomics approaches are required to determine the level of mixed BSH- and MSH-protein disulfides by combining the direct shotgun approach with OxICAT or the NEM-biotin switch assay coupled to Brx or Mrx1 (Leichert et al., 2008; Kehr et al., 2011). In addition, the regulatory roles for only few *S*-bacillithiolated and *S*-mycothiolated proteins have been studied thus far, including the redox regulator OhrR and the methionine synthase MetE (Lee et al., 2007; Chi et al., 2011). However, many interesting metabolic enzymes, redox-sensing transcription factors or virulence factors might be controlled by protein *S*-thiolations in the pathogenic bacteria *S. aureus* and *M. tuberculosis* that remain to be elucidated in future research. Thus, it is an exciting field for new frontiers of science to unravel the regulatory potential of emerging protein *S*-thiolations in bacteria.

### Conflict of interest statement

The authors declare that the research was conducted in the absence of any commercial or financial relationships that could be construed as a potential conflict of interest.
